# Real-Time Noninvasive Measurement of Glucose Concentration Using a Modified Hilbert Shaped Microwave Sensor

**DOI:** 10.3390/s19245525

**Published:** 2019-12-13

**Authors:** Levon Odabashyan, Arsen Babajanyan, Zhirayr Baghdasaryan, Seungwan Kim, Jongchel Kim, Barry Friedman, Jung-Ha Lee, Kiejin Lee

**Affiliations:** 1 Department of Radiophysics, Yerevan State University, Yerevan 0025, Armenia; levon.odabashyan@ysumail.am (L.O.); barsen@ysu.am (A.B.); zhirayr.baghdasaryan@ysumail.am (Z.B.); 2 Department of Physics, Sogang University, Seoul 121-742, Korea; seungwan74@hanmail.net (S.K.); kjchel00@riccimicrowave.com (J.K.); 3 Department of Physics, Sam Houston State University, Huntsville, TX 77341, USA; PHY_BAF@shsu.edu; 4 Department of Life Science, Sogang University, Seoul 121-742, Korea; jhleem@sogang.ac.kr

**Keywords:** real-time, glucose concentration, modified Hilbert, transmission parameters, microwave sensor, glucometer

## Abstract

We developed a microwave glucose sensor based on the modified first-order Hilbert curve design and measured glucose concentration in aqueous solutions by using a real-time microwave near-field electromagnetic interaction technique. We observed S_21_ transmission parameters of the sensor at resonant frequencies depend on the glucose concentration. We could determine the glucose concentration in the 0–250 mg/dL concentration range at an operating frequency of near 6 GHz. The measured minimum detectable signal was 0.0156 dB/(mg/dL) and the measured minimum detectable concentration was 1.92 mg/dL. The simulation result for the minimum detectable signal and the minimum detectable concentration was 0.0182 dB/(mg/dL) and 1.65 mg/dL, respectively. The temperature instability of the sensor for human glycemia in situ measurement range (27–34 °C for fingers and 36–40 °C for body temperature ranges) can be improved by the integration of the temperature sensor in the microwave stripline platform and the obtained data can be corrected during signal processing. The microwave signal–temperature dependence is almost linear with the same slope for a glucose concentration range of 50–150 mg/dL. The temperature correlation coefficient is 0.05 dB/°C and 0.15 dB/°C in 27–34 °C and 36–40 °C temperature range, respectively. The presented system has a cheap, easy fabrication process and has great potential for non-invasive glucose monitoring.

## 1. Introduction

Today, several diseases are causes of significant levels of mortality. The most significant one of them is diabetes mellitus that affects a tremendous number of people worldwide, and around half of them do not realize they have the disease. It is expected that in the future the rate of diabetics will increase [1,2,3]. The cause of this disease is an abnormal increase of glucose in the blood. Therefore diabetics need to test their glucose levels several times a day (for proper treatment) by using glucose sensors.

Presently, most glucose sensors are based on invasive measurement techniques that have a low price and provide high accuracy. However, these sensors can cause pain, are not comfortable for self-monitoring and the risk of infections is not eliminated. Due to the drawbacks mentioned above, medicine requires glucose sensors based on a non-invasive measurement technique [4,5,6].

A microwave sensor is presented here as the main part of a non-invasive glucometer. The sensor is based on the measurement of microwave response of a system due to the interaction of microwaves and materials. The wavelength of the microwave signal (3–30 cm for the 1–10 GHz range) allows the signal to easily penetrate many relevant biological materials (skin depth is up to a few mm) due to their low conductivity (up to 10 S/m). A measuring instrument based on microwaves has the ability to measure parameters inside a sample with a non-contact method, data which cannot be obtained invasively. At the same time, low data acquisition time (a few milliseconds) provides the detection and monitoring of solute (D-glucose, NaCl, hemoglobin) concentrations in complex mixtures in real-time with high sensitivity and accuracy. These parameters have a great role for investigations in biomedical and clinical testing [7,8]. Several types of glucose biosensors were developed for non-invasive glucose monitoring which are based on various techniques [9] such as a measurement of microwave response (shift in the resonance frequency, and transfer matrix) [10], electrochemical reaction [11], ultrasonic [12] and temperature [13] measurements, organic light-emitting devices [14], spectroscopic technique [15], bio-micro-electromechanical system [16], etc. However, they are limited to a material under test (MUT), are mainly invasive, and require improvement in accuracy and repetition. The microwave response technique is also applicable to discriminate the two types of chiral objects: the D- and L-glucose solutions [17].

In this paper, we propose a microwave sensor based on the modified Hilbert curve of the first order for the D-glucose (C_6_H_12_O_6_) concentration. The measurements are done on an aqueous solution by measuring the microwave transmission coefficient *S*_21_ at resonant frequencies of about 6 GHz. The change in transmission coefficient *S*_21_ is directly related to the change in the glucose concentration due to electromagnetic interaction between the microwave sensor and the samples. We selected the concentrations of glucose close to that found in human blood (from 50 mg/dL to 250 mg/dL) for both normal and diabetic patients. As a background, we chose deionized (DI) water.

The proposed sensor is optimized in order to obtain high sensitivity to the glucose concentration in a biological solution. We optimized the sensor to be sensitive to an amplitude change but not frequency because it is easier and less expensive to realize amplitude rather than frequency detection on a hardware level. The clinical prototype, based on our sensor platform, is possible to realize with low resources in the case of amplitude sensing. The measured parameter is the transmission coefficient *S*_21_ (but not reflection coefficient, *S*_11_, while *S*_11_ is more sensitive usually) because it is easier to provide precise measurement of transmission rather than reflection in a commercial device. We also modeled the Hilbert curve sensor by using high-frequency simulation software (HFSS) which accurately predicts the response of the microwave signal. The simulated results for *S* parameters of the sensor in both cases, without and with the material under test (MUT) were in good agreement with the obtained experimental data.

The methodology of non-invasive sensing of glucose mainly is based on the electromagnetic field–MUT interaction in the microwave (1–15 GHz) or near-infrared (800–1200 nm) regions [4,5,6,7,8,9,10,11,12,13,14,15,16]. Devices consist of the sensing element and the signal source/detector. Errors, noises or disturbance factors measurements are correlated by hardware control or by software signal processing procedures. Research now mostly concentrates on the improvement of the sensing element (sensor) and signal processing (for example, by using artificial intelligence), thus, the sensor has a high impact on the whole process. Therefore, this design of the sensor may be useful for researchers and engineers to improve the sensitivity of current glucometers and biosensors.

## 2. Materials and Methods

**Figure 1:**
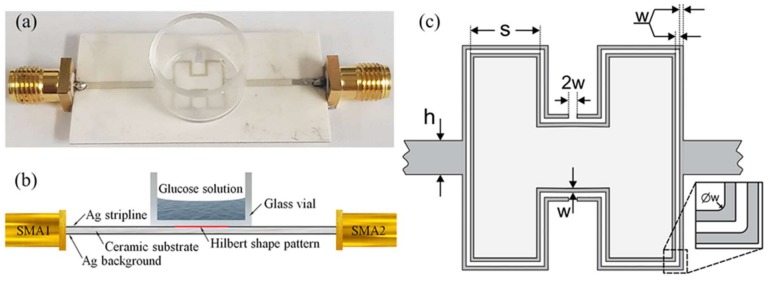
The modified 1st order Hilbert-shaped sensor with quartz vial: (**a**) optical image; (**b**) cross-sectional schematic structure; (**c**) structural geometry of sensor pattern.

Figure 1 shows (a) the optical and (b) structural cross-sectional image of the prepared modified 1st order Hilbert-shaped sensor with quartz vial and glucose solution. The ceramic substrate of the sensor was covered from both the upside and downside by a thin layer (about 50 μm) of silver paste and was dried out at a temperature of 100 °C for 20 min. After that, the sensor structure was patterned by using a laser patterning technique on one side. Finally, the sensor was annealed 130 min in 2 steps. In the first step, annealing is done for 600 °C for 60 min and the second, 880 °C for 70 min. Then a sensor was soldered to the conductors, with the center pin directly connected to the signal line and ground pins connected to the backside ground planes. The sensor pattern has a shape modeled as a modified Hilbert-shaped closed curve due to its space-filling and periodic (self-repeating) nature the Hilbert curve is one of the most recent novel geometries to be studied for antennas, filters, resonators in the microwave range. As a substrate, we used a ceramic with the dielectric permittivity of about 9.2 and dimensions of 20.4 mm × 40.4 mm × 1 mm. The geometry of the modified Hilbert-shaped sensor is shown in Figure 1c. Here *h*, *s*, and *w* are the width of the stripline, length of the Hilbert-shaped curve unit of the first order, and the width of the curve, respectively. The designed parameters of the sensor are *h* = 1 mm, *w* = 0.1 mm, and *s* = 2.6 mm.

The sensor with the MUT is connected to a vector network analyzer (VNA: Agilent E5071B) to analyze the transfer matrix of the system. At microwaves, various effects, such as ports and cable mismatch and frequency shift due to temperature and humidity, can affect measurement data. However, in a controllable measurement environment, these effects are repeatable and can be eliminated by the reference subtraction.

The resulting measurements were taken for 5 consecutive days and every day 10 times. The results presented in this article were obtained by averaging these data over the measurement results. For concentration and volume measurements each MUT was measured with an averaging factor of 5 by the VNA. The temperature measurements were done without any averaging factor.

In the experiment, we placed a quartz vial with aqueous solutions with various concentrations of D-glucose on the sensor and the response was measured. The dielectric constant of the quartz vial was 4.2, height was 8 mm, and the thickness was 1 mm. The outer and inner radius was 7.45 mm and 6.45 mm, respectively. The outer diameter of the vial was chosen to be 7.45 mm to achieve the complete interaction of the sensor with the MUT. The volume of the aqueous solution was kept at 500 µL during all experiments and in the simulation as well. Stabilized measurement results were obtained approximately 5 sec after the MUT was changed and high sensitivity can be achieved when the quartz vial was placed on the center of the sensing pattern, i.e., when the Hilbert curves symmetric center and the vial center are aligned. The resonator was calibrated with de-ionized (DI) water giving an *S*_21_ minimum of −25.3 dB. The data acquisition time for glucose real-time monitoring was 0.5 s and the ambient temperature was 25 °C. The entire system was placed on a mechanical vibration isolated table, and after each test, the quartz vial was washed and dried for the next MUT.

**Figure 2:**
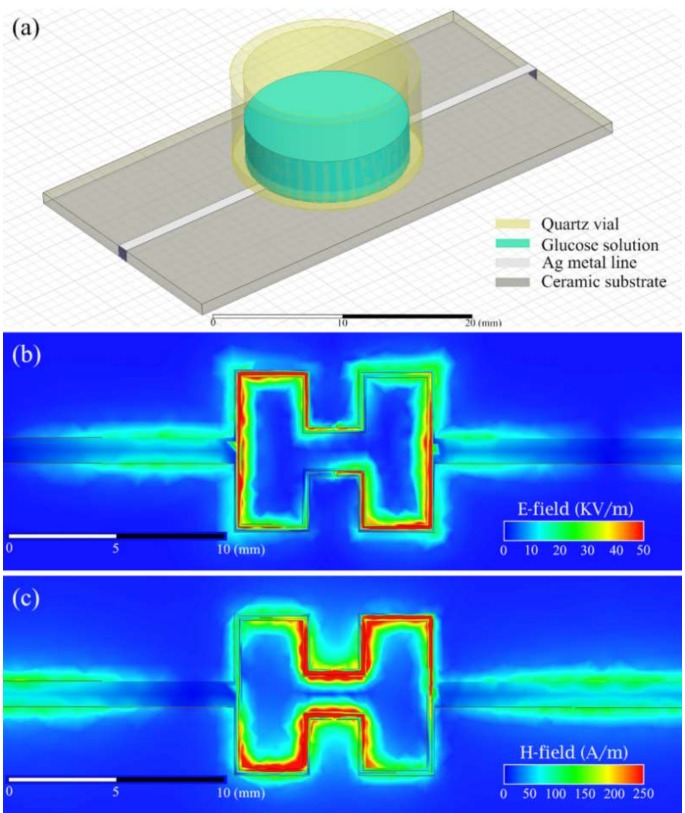
(**a**) The geometry of the sensor simulation model in high-frequency simulation software (HFSS). Simulated (**b**) electric and (**c**) magnetic near-field distribution at 6 GHz.

The geometry of the modified Hilbert-shaped biosensor was optimized by using HFSS software in order to obtain high sensitivity and resolution at fixed meta-element sizes by using the finite element method. To achieve more accurate simulation results, the upper side and downside of the substrate were set to be “Finite Conductivity”, and was chosen as a conductor, silver, with a thickness of 50 μm. The simulated results we obtained by applying the “Multipole Debye Model Input” function for the glucose solution to derive accurate results. The transmission coefficient *S*_21_ of the sensor was shifted due to the replacement of the MUT as a load. The geometry of the simulated model in HFSS and near-field electromagnetic field distribution are represented in Figure 2. The electromagnetic field intensity is concentrated around the sensing Hilbert-shaped pattern for both the electric field and the magnetic field distribution as shown in Figure 2b,c.

## 3. Theoretical Background

The operational principle of measurement is based on the change in microwave response of the testing device (transmission coefficient and system resonant frequency shift) due to changes in the complex dielectric permittivity of the MUT (for non-magnetic MUT). By measuring S parameters of the sensor (reflection coefficient S_11_ and transmission coefficient S_21_), we can indirectly evaluate the dielectric permittivity of MUT, which will provide valuable information about the electromagnetic characteristics of MUT. The relative permittivity of material has a complex form with $\varepsilon =\varepsilon^{\prime }-j\varepsilon^{\prime \prime }$, where $\varepsilon^{\prime }$ and *ε*″ are the real and imaginary parts of complex permittivity and causes electric energy storage and loss in the material, respectively.

On the other hand, the complex dielectric permittivity of solution depending on solute (glucose) concentration is given by $\varepsilon_{g}\left(\omega \right)=[\varepsilon_{0}^{\prime }\left(\omega \right)+c\delta^{\prime }]-j[\varepsilon_{0}^{\prime \prime }\left(\omega \right)+c\delta^{\;\prime \prime }$ where $\varepsilon_{0}\left(\omega \right)$ is the complex permittivity of DI water ($\varepsilon_{0}^{\prime }\left(\omega \right)$ = 73.89 and $\varepsilon_{0}^{\prime \prime }\left(\omega \right)$ = 21.55 at 6 GHz, 25 °C), c is the concentration of glucose, and *δ* is the increase in permittivity when the glucose concentration is raised by 1 unit ($\delta^{\prime }=0.00577$ (mg/dL)−1 and $\delta^{\prime \prime }=0.00015$ (mg/dL)−1) [18,19]. Here the complex dielectric permittivity of DI water is giving by the Cole–Cole relaxation model

(1) $$\varepsilon_{0}\left(\omega \right)=\varepsilon_{\infty }+\frac{\varepsilon_{s}-\varepsilon_{\infty }}{1+{\left(i\omega \tau \right)}^{1-\alpha }},$$

where $\varepsilon_{\infty }=8.7$ is the permittivity in the high-frequency limit, $\varepsilon_{s}=81$ is the static, low-frequency permittivity, τ=8.7 ns is characteristic relaxation time of DI water, ω is the angular frequency, and 0 < α < 1 is the exponent parameter. Note that for α=0 Debye relaxation model has an important approximation to study the effect of the glucose concentration on permittivity [20]. Due to the Monosaccharide molecule’s chemical structure, the viscous effect increases as the concentration of the glucose solution increases, resulting in increased relaxation times and correspondingly decreased dielectric constants and increased loss factor according to Equation (1). As a consequence, the maximum difference between *S*_21_ transmission coefficients of the sensor during the experiment is expected when the glucose level in the glucose sample is minimized and maximized at 0 (DI water) and 250 mg/dL (glucose–water solution) [21].

**Figure 3:**
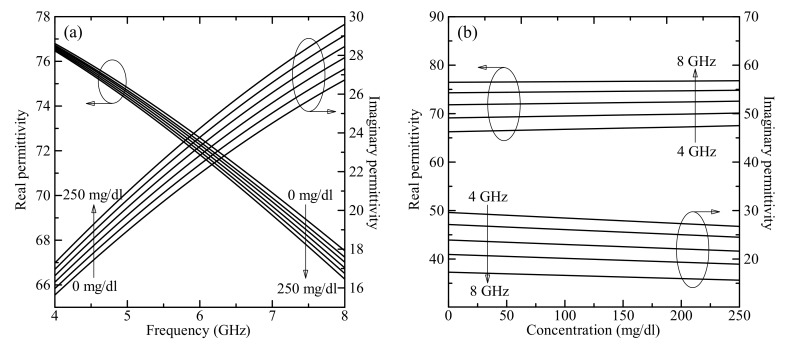
(**a**) Calculated real (left axis) and imaginary (right axis) part of the complex relative dielectric permittivity plotted as a function of the frequency for glucose aqueous solution with concentrations of 0 mg/dL to 250 mg/dL. (**b**) The dependences of the real (left axis) and imaginary (right axis) parts of the complex relative permittivity on the glucose concentration for the frequency range 4–8 GHz.

Figure 3a shows the dependence of real (left axis) and imaginary (right axis) parts of complex relative dielectric permittivity of D-glucose versus frequency for DI water and for glucose concentrations ranging from 0 mg/dL to 250 mg/dL. Figure 3b shows estimated real and imaginary parts of the complex relative dielectric permittivity of the D-glucose in the frequency range 4–8 GHz plotted as a function of the glucose concentration up to 250 mg/dL.

By increasing the operation frequency, the real part of permittivity shows decreasing behavior and the imaginary part shows increasing behavior, whereas by increasing the glucose concentration in the solution the real part of permittivity of the solution increases and the imaginary part decreases. Note that in our test the change in magnetic permeability was negligible since it is close to the magnetic permeability of free space for most biological materials [22].

## 4. Results and Discussion

**Figure 4:**
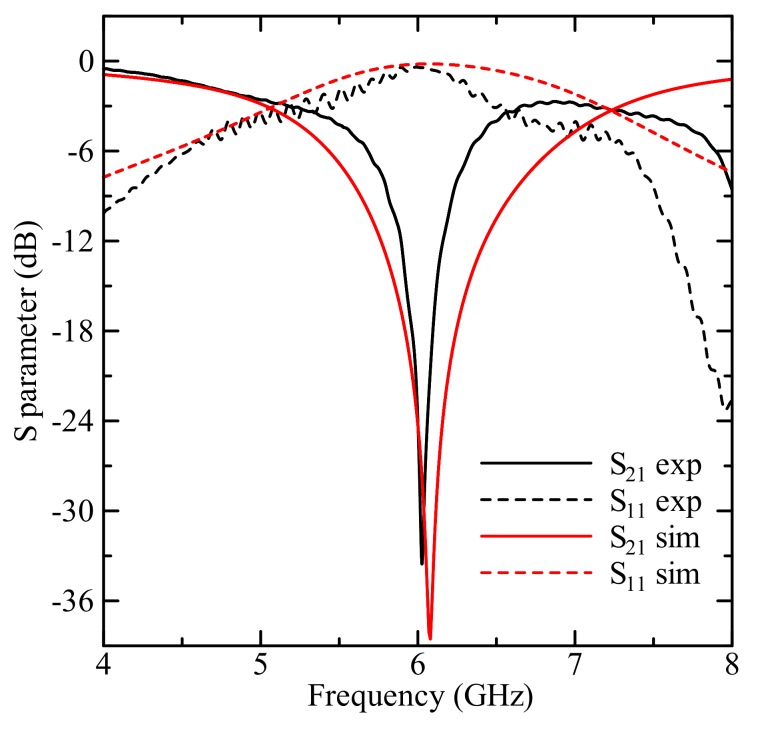
The experimental and simulated reflection/transmission coefficients of the sensor.

The (a) measured and (b) simulated transmission parameters for the designed sensor are presented in Figure 4. The resonant frequency is nearly 6 GHz for both measured and simulated data. The sensor has a Q-factor of about 62 for S_21_. With the loaded MUT (DI water) Q-factor decreased to 3.2 or by 95%. The shift between the measured and simulated resonant frequency of about 50 MHz and about 5 dB can be caused by port mismatching and cable losses, etc. In general, the behaviors of measured and simulated transmission parameters are in good agreement and described the testing process well.

**Figure 5:**
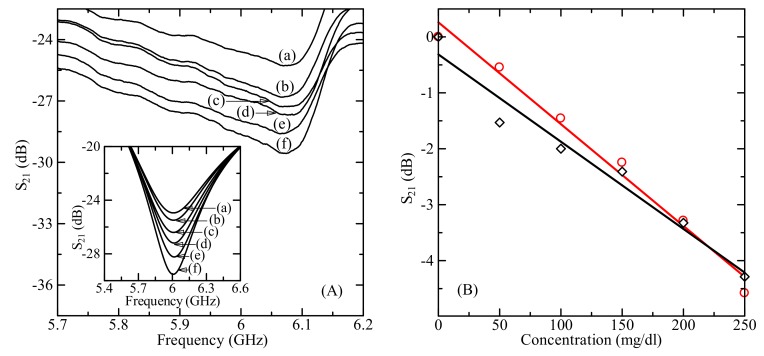
(**A**) Measured and simulated (inset) results of microwave transmission coefficient *S*_21_ profile for (a) DI water and for glucose aqueous solution with D-glucose concentration of (b) 50 mg/dL, (c) 100 mg/dL, (d) 150 mg/dL, (e) 200 mg/dL, and (f) 250 mg/dL with 500 µL volume. (**B**) The measured and simulated microwave response dependence on D-glucose concentration at the resonant frequency of about 6 GHz.

Loading the sensor with MUT causes the change of scattering parameters (resonant frequency, amplitude) due to the change of the total impedance of the system. Figure 5A shows the experimental and simulated results of microwave transmission coefficient *S*_21_ profiles for (a) DI water and for D-glucose aqueous solution with glucose concentration of (b) 50 mg/dL, (c) 100 mg/dL, (d) 150 mg/dL, (e) 200 mg/dL, and (f) 250 mg/dL. Both the experimental and the simulated curves have the resonant minimum value of S_21_ at the frequency around 6 GHz (6.07 GHz vs. 6.01 GHz). The transmission parameter *S*_21_ shows monotonously decreasing behavior as the glucose concentration increased as shown in Figure 5B for both the experiment and simulation. The maximum difference of *S*_21_ parameter for sensor loaded by DI water and solution with 250 mg/dL glucose concentration was about 4.3 dB at 6.07 GHz from experiment and 4.6 dB at 6.01 GHz from the simulation.

The relationship between *S*_21_ and glucose concentration is linear and the *S*_21_ trend varies with a slope of 0.0156 dB/(mg/dL) in measurement and 0.0182 dB/(mg/dL) in simulation. Stable linear relationships are important for detection and analyzing glucose concentrations in solution. The minimum detectable concentration *c_min_* of the designed sensor is defined as

(2) $$c={\frac{S^{R}}{\Delta S_{21}/\Delta c}}_{min}$$

where Δ*c* is the concentration variation, Δ*S*_21_ is the change in *S*_21_ corresponding to Δ*c*, and $S^{R}=0.03$ dB is the resolution of the experimental system. The minimum detectable concentration was found to be 1.92 mg/dL and 1.65 mg/dL for experiment and simulation, respectively (data are summarized in Table 1).

**Figure 6:**
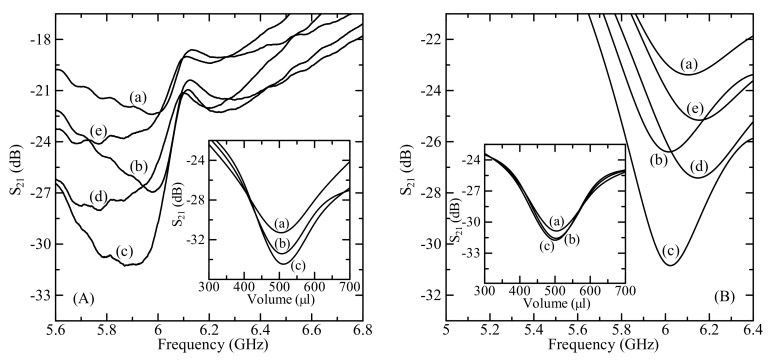
(**A**) Measured and (**B**) simulated microwave transmission coefficient *S*_21_ profiles for material under test (MUT) with volume of (a) 300 μL, (b) 400 μL, (c) 500 μL, (d) 600 μL, and (e) 700 μL at an operating frequency of about 6 GHz. The D-glucose concentration was 100 mg/dL. The inset shows the (**A**) measured and (**B**) simulated transmission coefficient *S*_21_ as a function MUT volume for D-glucose concentration of (a) 100 mg/dL, (b) 200 mg/dL, and (c) 250 mg/dL.

The transmission coefficient *S*_21_ of glucose solution also depended on the volume of solution. Figure 6 shows the microwave transmission coefficient *S*_21_ profile of aqueous glucose solution for MUT volume ranging from 300–700 µL with an interval of 100 µL. The concentration of glucose solution was 100 mg/dL.

As we can see from Figure 6, when the volume of glucose–water solution was increased, the transmission coefficient decreased in the range up to 500 µL and increased after 500 µL. Because the measurement sensitivity is the highest at 500 µL volume of the MUT (inset of Figure 6), all measurements and simulations were done at 500 µL volume. Note that the detection sensitivity in experiments was about 3.3 times higher than that for simulation at 500 μl (−0.0204 dB/(mg/dL) vs. −0.0062 dB/(mg/dL)) while at 400 μl MUT volume the detection sensitivities was different by about 2.46 times (0.0037 dB/(mg/dL) vs. −0.0015 dB/(mg/dL)).

**Figure 7:**
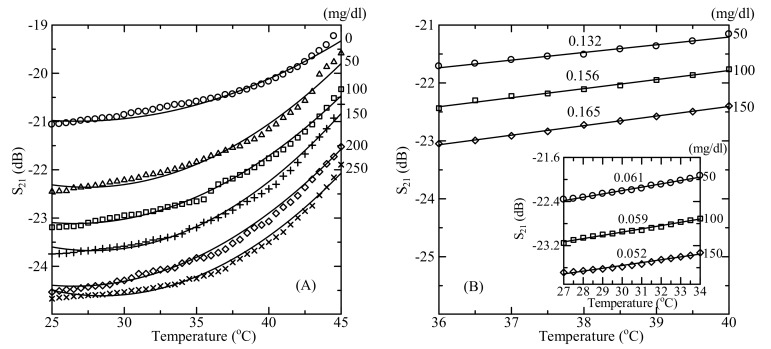
(**A**) Measured microwave transmission coefficient *S*_21_ plotted as a function of the MUT temperature for different D-glucose concentrations at the resonant frequency near 6 GHz. (**B**) Measured *S*_21_ plotted as a function of the MUT temperature for 50, 100, 150 mg/dL D-glucose concentrations at 6 GHz in 36–40 °C and 37–34 °C (inset figure) ranges.

The microwave response of the sensor is sensitive to the change of the MUT temperature as well. Figure 7A shows the dependence of measured microwave transmission coefficient minima *S*_21_ versus temperature for the DI water and D-glucose aqueous solution with a glucose concentration range of 50–250 mg/dL at 6 GHz. The MUT volume was 500 μl. When the temperature of the glucose solution increased from 25 °C to 45 °C, the transmission coefficient *S*_21_ increased due to the decrease of the relative permittivity of glucose solution [23]. The temperature influence of *S*_21_ of the microwave sensor was slight at low temperatures (until 35 °C) while for the high temperatures *S*_21_ increase more rapidly as shown in Figure 7. The linear slope of this dependence is 0.22 dB/°C vs. 0.006 dB/°C (about 3.5 times), 0.24 dB/°C vs. 0.005 dB/°C (about 4.5 times), and 0.23 dB/°C vs. 0.004 dB/°C (about 6 times) for the 50 mg/dL, 150 mg/dL, and 250 mg/dL at the high and low temperatures, respectively.

The temperature variation by 1 °C near 25 °C for 100 mg/dL glucose concentration is 0.0169 dB. There is about the same value of shifts in the signal that occurs if glucose concentration changes by 1.08 mg/dL which is half of the minimum detectable signal—1.92 mg/dL. In our measurements, the temperature was kept at 25 ± 0.1 °C (this is equivalent to glucose variation of 0.08 mg/dL) that allows us to neglect temperature influence. While, the temperature variation by 7 °C in the 27–34 °C range (this is the comfort feeling range for finger temperature [24]) for 100 mg/dL glucose concentration is 0.4151 dB with a slope of 0.156 dB/°C (Figure 7B). The signal shifts by the same value if the glucose concentration changes by 6.5 mg/dL which is 3.4 times bigger than the minimum detectable signal. Finally, the temperature variation by 0.5 °C in the 36–40 °C range (the value change of the human body normal temperature during the day [25,26]) for 100 mg/dL glucose concentration is 0.0754 dB with a slope of 0.059 dB/°C (inset of Figure 7B). The signal shifts by the same value if the glucose concentration changes by 4.85 mg/dL which is 2.5 times bigger than the minimum detectable signal. Thus, the detection resolution of 1.92 mg/dL is not possible without temperature correction in the 27–34 °C and the 36–40 °C temperature ranges. However, this correction is easy due to the linear dependence of the microwave signal on the temperature in the discussed temperature range. The instability caused by the temperature variation can be finally corrected by the correlation of the sensor temperature response behavior. The microwave signal–temperature dependence is almost linear with the same slope for a glucose concentration range of 50–150 mg/dL. The temperature correlation coefficient is 0.05 dB/°C and 0.15 dB/°C in the 27–34 °C and the 36–40 °C temperature range, respectively. A temperature sensor can be simply integrated in the microwave stripline platform and the obtained data can be corrected during signal processing. In particular, by using a digital temperature sensor and positioning finger-print sensor it is possible to made temperature and volumetric correction of obtained microwave data to get proper sensitivity and accuracy for glucose detection in the 0–250 mg/dL concentration range.

In laboratory conditions, with a fixed volume (500 ± 10 µL) and temperature (25 ± 1 °C) of MUT the repeatability of measurements is very high with an accuracy of about 98%. For in-situ measurements, the instability caused by these two parameters must be corrected by correlation of sensor volume and temperature response behaviors.

**Figure 8:**
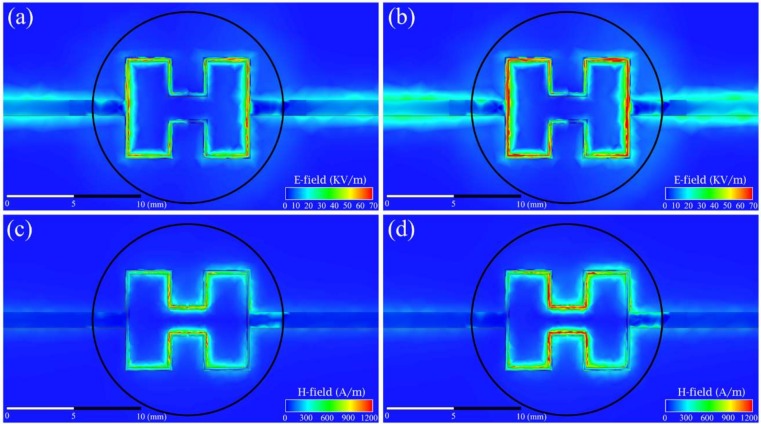
The simulated images for the (**a**,**b**) electric and (**c**,**d**) magnetic field distributions for electromagnetic interaction between the sensor and MUT with (**a**,**c**) 0 mg/dL (DI water) and (**b**,**d**) 250 mg/dL D-glucose aqueous solution, respectively. The solid black circle shows the location of the MUT.

We also simulated the electromagnetic field interaction between the sensor and the glucose solution, to visualize the electromagnetic field distribution during sensor/MUT interaction. Figure 8 shows the simulated images for the (a), (b) electric (E) and (c), (d) magnetic (H) field distributions for electromagnetic interaction between the sensor and the MUT with (a), (c) 0 mg/dL (DI water) and (b), (d) 250 mg/dL D-glucose aqueous solution, respectively. The black circle indicates the location of the MUT. When the glucose concentration increased, both electric and magnetic fields strengths increased too. Changes in electromagnetic field distribution were due to the increase in the dielectric permittivity of glucose solution, when the concentration of glucose in solution was increased. MUT response on the E-field is stronger than that of the H-field as shown in Figure 8. However, there is some change in magnetic field distribution depending on glucose concentration due to the imaginary part (i.e., conductivity) of the complex dielectric permittivity of the MUT. Note that the change in conductivity is in order of 0.6 S/m for 250 mg/dL glucose concentration change. In the case of ionic fluids and mixtures (NaCl, KCl, etc.), this change will be stronger and essential. The microwave magnetic field induces surface currents in the conductive medium which dissipates as heat and changes the total distribution in the H-field of the sensor.

## 5. Conclusions

The microwave sensor based on modified Hilbert-shaped closed curve was designed and prepared as a non-invasive glucometer for monitoring of glucose concentrations in glucose aqueous solution.

The sensor is able to determine glucose concentrations with a non-destructive method. The linear relationship between measured *S*_21_ response and D-glucose concentrations at about 6 GHz was found to be 0.0156 dB/(mg/dL). The minimum detectable resolution for glucose concentration was about 1.92 mg/mL for 500 µL MUT volume.

The temperature instability of the sensor can be corrected by the temperature correlation coefficient of 0.05 dB/°C and 0.15 dB/°C in 27–34 °C and 36–40 °C temperature range, respectively.

The results show the potentiality of a sensor for glucose monitoring. This microwave biosensor has been especially successful for the real-time monitoring of the concentration of glucose in aqueous solutions and it may serve as a platform to develop a bloodless glucometer.

## Figures and Tables

**Table 1:** The main parameters of the designed sensor.

Sensor Parameter	Measurement	Simulation
Dynamic range for *S*_21_	4.3 dB	4.6 dB
Resonant frequency	6.07 GHz	6.01 GHz
Sensitivity for *S*_21_	0.0156 dB/(mg/dL)	0.0182 dB/(mg/dL)
Minimum detectable concentration	1.92 mg/dL	1.65 mg/dL
